# Retinal Carotenoid Supplementation Increases HDL Cholesterol in Humans and Mice

**DOI:** 10.3390/life16010023

**Published:** 2025-12-23

**Authors:** Binxing Li, Emmanuel K. Addo, Fu-Yen Chang, Shukui Guo, Moses Awuni, Emily Conway, Jialai Ying, Dylan Ramos, Paul S. Bernstein

**Affiliations:** 1 Department of Ophthalmology and Visual Sciences, John A. Moran Eye Center, University of Utah, 65 Mario Capecchi Drive, Salt Lake City, UT 84132, USA; 2 Department of Nutrition and Integrative Physiology, University of Utah, Salt Lake City, UT 84132, USA

**Keywords:** carotenoid, lutein, zeaxanthin, HDL, cholesterol, triglyceride

## Abstract

Carotenoid supplementation may reduce the risk of age-related macular degeneration (AMD). These retinal nutrients are hydrophobic molecules obtained from the diet that are transported to the retina through high-density lipoprotein (HDL) complexes. HDL cholesterol is a recognized biomarker for AMD risk. This study examined the effect of carotenoid supplementation on circulating HDL cholesterol levels. Serum lipid profiles were measured in 20 participants from the Lutein and Zeaxanthin in Pregnancy (L-ZIP) trial, which enrolled 40 pregnant women. In addition to standard prenatal supplements, half received 10 mg of lutein and 2 mg of zeaxanthin daily from the first trimester, and half received a placebo. Carotenoid supplementation significantly increased HDL cholesterol in the third trimester, with no changes in total cholesterol, LDL cholesterol, or triglycerides (TG) across trimesters. To further evaluate individual carotenoids, serum lipids were analyzed in macular pigment transgenic mice fed lutein, zeaxanthin, or β-carotene for one month. All three carotenoids significantly increased HDL cholesterol and reduced TG levels, with the effect ranking as zeaxanthin > lutein > β-carotene. These findings suggest that carotenoid supplementation modulates the serum lipid profile—elevating HDL cholesterol and lowering TG—which may contribute to protection against AMD and other lipid-associated diseases.

## 1. Introduction

Carotenoids are natural antioxidants that can quench reactive oxygen species (ROS), thereby lowering oxidative stress and reducing inflammation [1,2,3,4]. Lutein, zeaxanthin, and β-carotene are the major dietary carotenoids detected in the human retina [5,6,7]. Among these, lutein and zeaxanthin accumulate in the macular region at very high concentrations and are referred to as macular pigments, whereas β-carotene is present only in trace amounts. β-Carotene also serves as the precursor to the visual chromophore 11-*cis*-retinal [8,9]. Many clinical trials, including the Age-Related Eye Disease Study 1 and 2 (AREDS1 and AREDS2), have suggested that supplementation with retinal carotenoids reduces the risk of retinal diseases such as age-related macular degeneration (AMD) [10,11,12,13,14].

Humans and animals cannot synthesize carotenoids because they lack the enzymes required for carotenoid biosynthesis; therefore, these nutrients must be obtained from their diet [15,16]. Carotenoids are abundant in green leafy vegetables and orange or yellow-colored fruits. Being hydrophobic, carotenoids are transported in the body via lipoproteins, similar to most lipids and cholesterol [17,18].

Genome-wide association studies (GWAS) have revealed that genetic variants of several HDL pathway proteins—such as ABCA1, SR-BI, LIPC, and CETP—are significantly associated with retinal carotenoid levels [19,20]. Upregulation of the HDL receptor SR-BI increases carotenoid levels in the liver and eyes of mice [21], while deletion of ApoA1, the main apolipoprotein of HDL, raises hepatic carotenoids but lowers retinal carotenoids in macular pigment mice [22]. Together, these findings indicate that carotenoids are transported through the HDL pathway, particularly from the liver to the eye.

Although retinal carotenoids are likely transported by HDL particles, they are also found in LDL particles in human serum [23,24]. Carotenes lacking oxygen atoms, such as α- and β-carotene, preferentially bind to LDL particles, whereas oxygen-containing xanthophylls such as lutein and zeaxanthin preferentially associate with HDL particles. About 15–20 carotenoids are detected in human serum, yet only lutein and zeaxanthin are delivered to the retina [25,26]. Thus, the transport of macular carotenoids appears to be a selective process, akin to HDL’s reverse cholesterol transport.

HDL cholesterol is strongly linked to both coronary artery disease (CAD) and AMD [27,28,29]. Many studies have explored the relationships between carotenoids and AMD and between HDL cholesterol and AMD. However, relatively few have examined how HDL cholesterol and carotenoids interact directly, leaving some questions about their potential protective roles in AMD and other diseases open to further study.

Here, we examined the effects of carotenoid supplementation on serum lipid profiles in humans and mice using HPLC and a lipid analyzer. We found that carotenoid intake increased HDL cholesterol and decreased triglyceride levels in both species.

## 2. Materials and Methods

### 2.1. L-ZIP Clinical Trial

The Lutein and Zeaxanthin in Pregnancy (L-ZIP) clinical trial was conducted at the Moran Eye Center, University of Utah (Salt Lake City, UT, USA), between September 2019 and January 2022 [30]. Detailed methods and findings are reported elsewhere (ClinicalTrials.gov Identifier: NCT03750968). Briefly, the L-ZIP trial was a single-site, prospective, randomized, double-masked, active-controlled study designed to assess whether prenatal carotenoid supplementation affects biomarkers of maternal and infant carotenoid status or whether the third trimester represents a period of maternal carotenoid depletion. The study adhered to all regulatory and ethical requirements and was approved by the University of Utah Institutional Review Board.

A total of 47 low-risk pregnant women (≥18 years old; 41 completed this study) were enrolled during the first trimester (≤14 weeks of gestation), and randomized (1:1) to either the carotenoid supplementation group (*n* = 21) or the control group (*n* = 20). The carotenoid group received a daily softgel containing 10 mg lutein and 2 mg zeaxanthin in safflower oil (Kemin Health L.C., Des Moines, IA, USA), while the control group received a matching placebo containing safflower oil only. The dosage of our supplement might be slightly higher than natural dietary carotenoid intake; however, it remains within the established biosafety range [17,31]. Supplements were taken once daily with a meal to enhance absorption, beginning in the first trimester and continuing through delivery (~7 months). Participants were instructed to avoid other carotenoid-containing supplements during the study. Compliance was monitored by monthly check-ins, text reminders, and pill counts at each visit.

Blood samples were collected for serum preparation as previously described [30], then transferred to labeled microtubes, and stored at –80 °C until analysis. In this study, 10 serum samples per group were randomly selected for lipid profile assessment at baseline (T1), second trimester (T2, 22–26 weeks), and third trimester (T3, 37–39 weeks).

### 2.2. Carotenoid Detection by HPLC

Serum carotenoids were extracted by mixing 200 μL of serum with ethanol containing 0.1% butylated hydroxytoluene (BHT) to precipitate proteins, followed by extraction with ethyl acetate. After vortexing and centrifugation (2000× *g*, 5 min), the organic phase was collected and pooled across extractions. The extract was evaporated under nitrogen, reconstituted in a methanol/methyl tert-butyl ether (80:20 *v*/*v*) mobile phase, centrifuged at 12,000× *g* for 10 min, and analyzed using an Agilent 1260 HPLC system equipped with a YMC C30 carotenoid column, as reported [32,33]. Detection was performed at 450 nm using a diode-array detector at a flow rate of 1 mL/min over 30 min.

### 2.3. Animal Carotenoid-Feeding Experiments

Macular pigment transgenic mice (*RPE-Cre/Bco2*^−/−^) were recruited for this carotenoid-feeding experiment, mainly due to their background of the deficiency of xanthophyll carotenoid cleavage enzyme BCO2, mimicking the carotenoid metabolism in the human body, as well as the availability of the animal. This mouse strain was generated by crossing the *Bco2* knockout mice (gift from Dr. Johannes von Lintig at Case Western Reserve University) into a strain with Cre expressed specifically in the retinal pigment epithelium (RPE) (gift from Dr. Christian Grimm at the University of Zurich). All mice were identified using genotyping methods previously reported [34,35,36]. Male and female animals were randomly chosen for this experiment.

Twelve 3-month-old macular pigment transgenic mice were fed vitamin A-deficient chow for 1 month to increase carotenoid bioavailability. Mice were then divided into four groups (*n* = 3/group) and fed with zeaxanthin, lutein, β-carotene, and the control placebo chow for one month (DSM ActiLease^®^ beadlets-carotenoid (DSM Nutritional Products, Basel, Switzerland), mixed into the base diet at a ratio of 1 g/kg by TestDiet (Richmond, IN, USA)). After one month of feeding on their respective chow diet, these mice were anesthetized with isoflurane (2%). Subsequently, their blood samples were collected into 1.3 mL Serum-Z Micro Tubes (#2071721, SARSTEDT, Nümbrecht, Germany) and centrifuged at 1000× *g* for 5 min at room temperature to prepare the serum. The serum was kept in −80 °C freezer until lipid and carotenoid analysis.

All animal procedures were approved by the Institutional Animal Care and Use Committee of the University of Utah (ICAUC of UU) and conformed to the standards in the ARVO Statement for the Use of Animals in Ophthalmic and Vision Research.

### 2.4. Measurement of Serum Lipid Profiles

Lipid profiles in the serum of humans and mice were measured using a lipid analyzer. Briefly, 35 µL of serum were loaded onto a lipid test strip from The Sommet, Inc. (#98LS10C, Irvine, CA, USA) and tested on A CURO L7 lipid analyzer purchased from O2 Lifecare, Inc. (Irvine, CA, USA) at room temperature. The values of total cholesterol, HDL cholesterol, LDL cholesterol, and triglycerides were then recorded. The measurements of serum lipid profile were repeated three times per sample. In addition, if the value of serum lipid is too high, it would be diluted with Corning™ Cell Culture Phosphate-Buffered Saline (1×) (MT21040CV, Fisher Scientific (Waltham, MA, USA)) and adjusted into the recommended range for accuracy. The lipid profiles of mice fed with carotenoids and several patients were measured using this method.

### 2.5. Statistical Analysis

During our data analysis, continuous variables, including serum carotenoids and lipid parameters (total cholesterol, LDL cholesterol, HDL cholesterol, and triglycerides), were summarized as mean ± standard deviation (SD). Between-group comparisons were performed using independent two-sample t-tests after confirming normality and equal variances. Because randomization produced no significant baseline differences and the groups were comparable in age, sex, BMI, and baseline lipid levels, covariate adjustment was not required. Associations between carotenoid and lipid parameters were examined using Pearson correlation coefficients after confirming normality and linearity. Corresponding scatter plots with regression lines were generated to visualize these relationships. All analyses were conducted in STATA, and figures were prepared using GraphPad Prism 8. Statistical significance was defined as a two-tailed *p* < 0.05.

## 3. Results

To investigate whether carotenoid supplementation alters serum lipid composition, we measured carotenoid and lipid profiles in adult participants of the Lutein and Zeaxanthin in Pregnancy (L-ZIP) clinical trial. Pregnant women were enrolled during the first trimester and received a daily gel capsule containing 10 mg lutein and 2 mg zeaxanthin or a placebo until the end of the third trimester.

**Figure 1:**
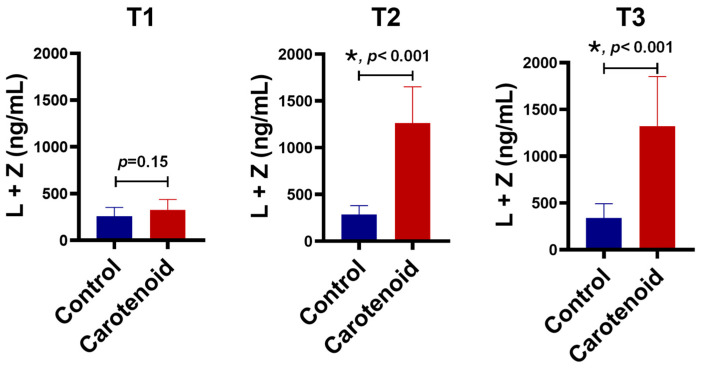
Contents of lutein and zeaxanthin in human serum detected by HPLC. Pregnant women were enrolled in this clinical trial in the first trimester (T1). Supplementation of 10 mg lutein and 2 mg zeaxanthin significantly increased serum carotenoids in the second trimester (T2) and the third trimester (T3). *n* = 10 subjects/group; * *p* < 0.001.

Figure 1 shows the serum concentrations of lutein and zeaxanthin across the three trimesters. The baseline carotenoid concentration was approximately 300 ng/mL, with no significant difference between the supplementation and control groups. After ~3 months of carotenoid administration (second trimester), serum carotenoids in the supplementation group increased approximately 4.4-fold relative to controls. Following ~6 months of supplementation (third trimester), carotenoid concentrations further rose to ~1330 ng/mL—about four times higher than controls. A slight but consistent increase was also observed from the first to the third trimester in the control group.

**Figure 2:**
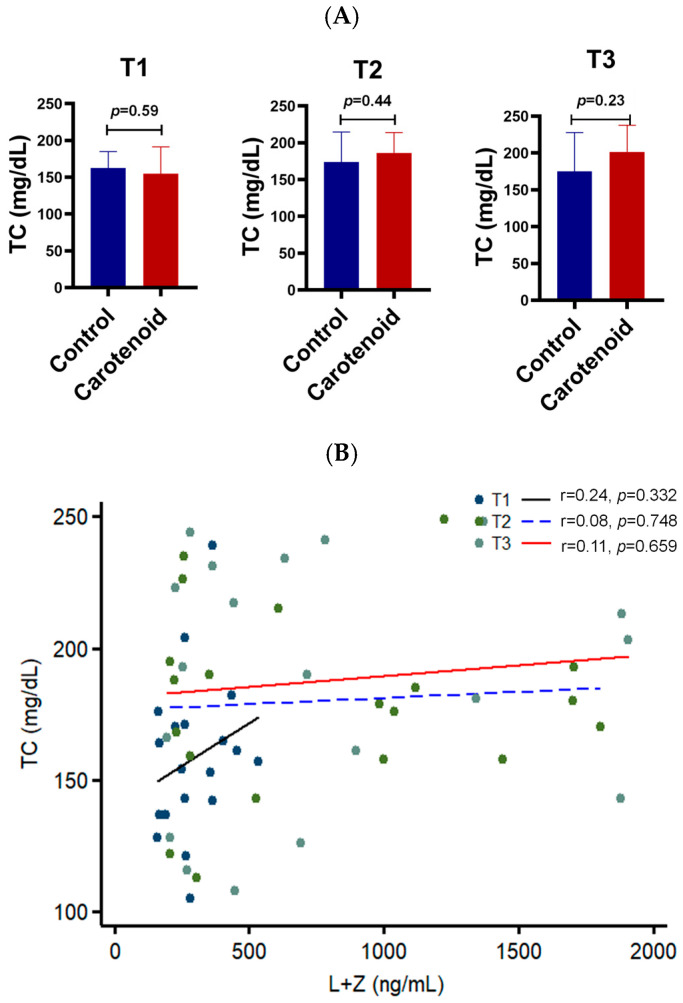
Effect of carotenoid intake on the total cholesterol in the human serum. No significant correlation is found between serum carotenoids and total cholesterol. (**A**) Total cholesterol (TC) in the serum of pregnant women. T1, first trimester; T2, second trimester; T3, third trimester. *n* = 10 subjects/group. (**B**) Correlation between serum carotenoids and total cholesterol. T1, black solid line; T2, blue dashed line; T3, red solid line.

Total cholesterol is a known risk factor for atherosclerosis, cardiovascular disease, and neurodegenerative disorders. It also reflects the overall status of lipoprotein particles in the serum. To determine whether carotenoid intake affects total cholesterol, we measured serum cholesterol levels using a lipid analyzer (Figure 2). No significant difference was observed between the carotenoid and control groups, with total cholesterol levels ranging between 150 and 200 mg/dL across all trimesters. Correlation analysis between serum carotenoids and total cholesterol yielded *R*-values of 0.24, 0.08, and 0.11 for the first, second, and third trimesters, respectively, none of which reached statistical significance. These results suggest that macular carotenoid intake does not alter total cholesterol levels.

**Figure 3:**
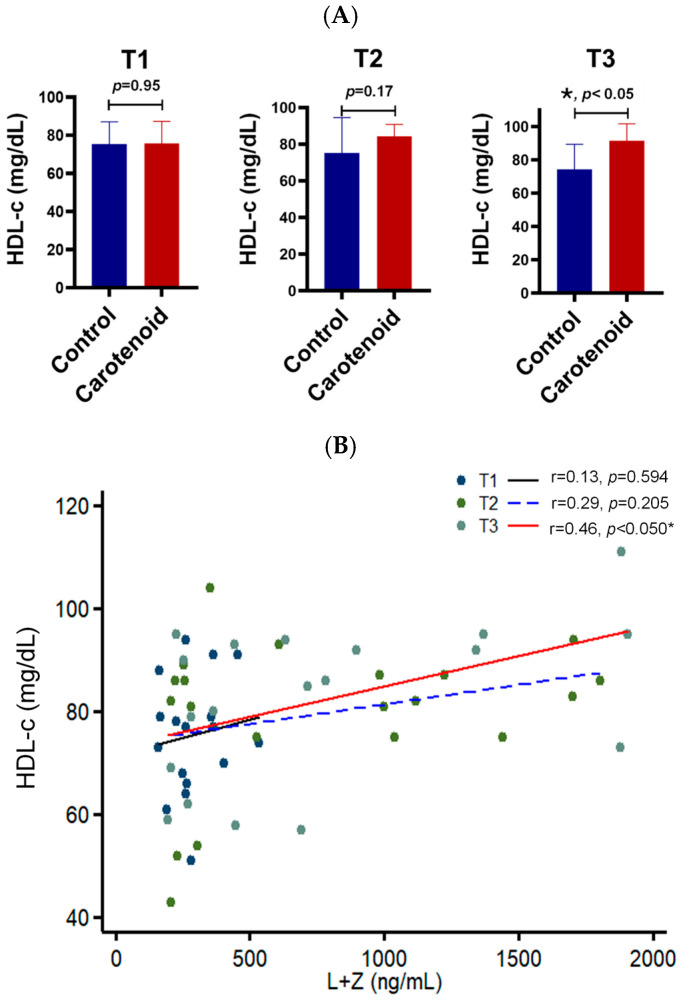
Effect of carotenoid intake on the HDL cholesterol in the human serum. Carotenoid intake increases HDL cholesterol, and the serum carotenoids are significantly correlated with serum HDL cholesterol in the third trimester. (**A**) HDL cholesterol (HDL-c) in the serum of pregnant women. T1, first trimester; T2, second trimester; T3, third trimester. *n* = 10 subjects/group; * *p* < 0.05. (**B**) Correlation between serum carotenoids and HDL cholesterol. T1, black solid line; T2, blue dashed line; T3, red solid line. * *p* < 0.05.

HDL cholesterol—commonly referred to as “good cholesterol”—represents cholesterol carried by HDL particles in serum and serves as a protective biomarker against atherosclerosis, diabetes, AMD, and other diseases. Our data show that HDL cholesterol levels in these pregnant women remained within the healthy range (75–91 mg/dL) across all trimesters (Figure 3). Compared with the placebo group, carotenoid supplementation increased HDL cholesterol by approximately 1.12-fold in the second trimester and 1.23-fold in the third trimester. Correlation analyses revealed that the *R*-value between HDL cholesterol and carotenoids increased from 0.13 in the first trimester to 0.46 in the third trimester, becoming statistically significant at that stage. These findings indicate that lutein and zeaxanthin supplementation significantly elevates HDL cholesterol in human serum.

**Figure 4:**
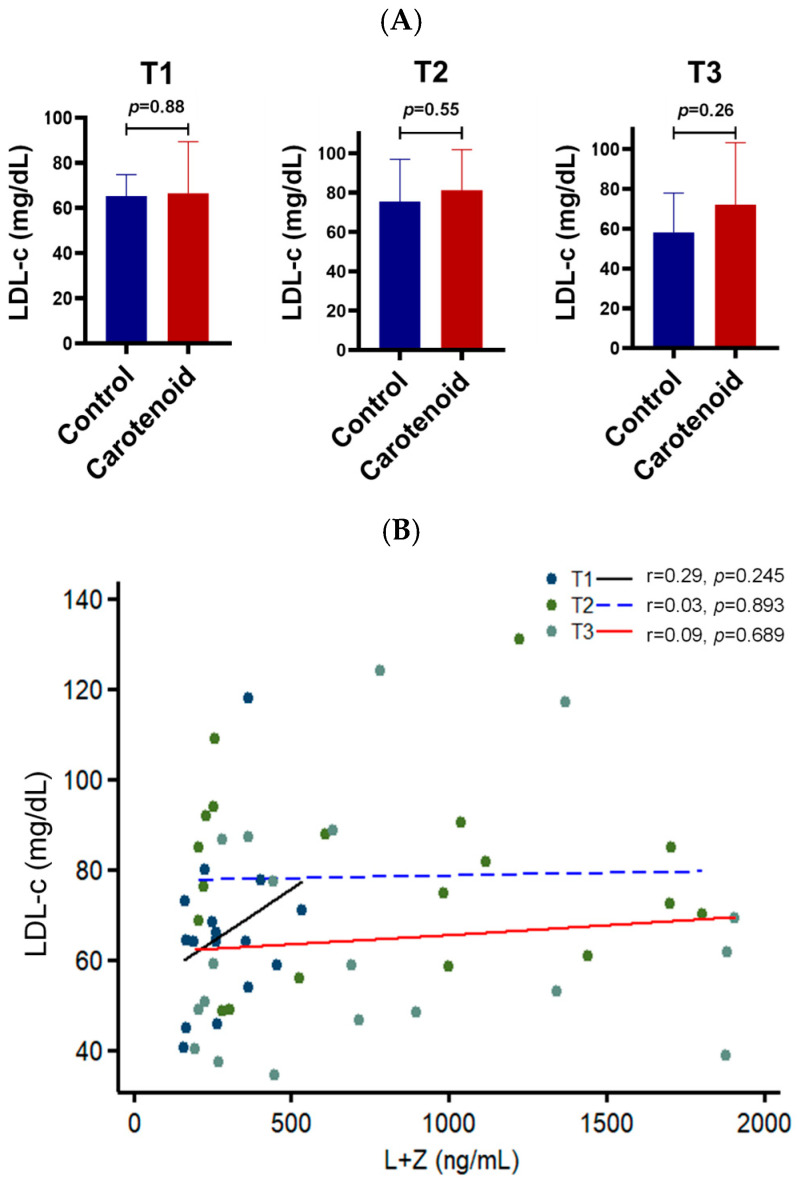
Effect of carotenoid intake on the LDL cholesterol in the human serum. No significant correlation is found between serum carotenoids and LDL cholesterol. (**A**) LDL cholesterol (LDL-c) in the serum of pregnant women. T1, first trimester; T2, second trimester; T3, third trimester. *n* = 10 subjects/group. (**B**) Correlation between serum carotenoids and LDL cholesterol. T1, black solid line; T2, blue dashed line; T3, red solid line.

Next, we examined LDL cholesterol levels. LDL, or “bad cholesterol,” reflects the cholesterol bound to LDL particles and is associated with cardiovascular and liver diseases. As shown in Figure 4, no significant differences in LDL cholesterol were detected between subjects with or without carotenoid supplementation, with mean values ranging from 65 to 72 mg/dL. Correlation analysis showed *R*-values of 0.29, 0.03, and 0.09 across the three trimesters, none of which reached statistical significance, suggesting carotenoid supplementation does not affect LDL cholesterol.

**Figure 5:**
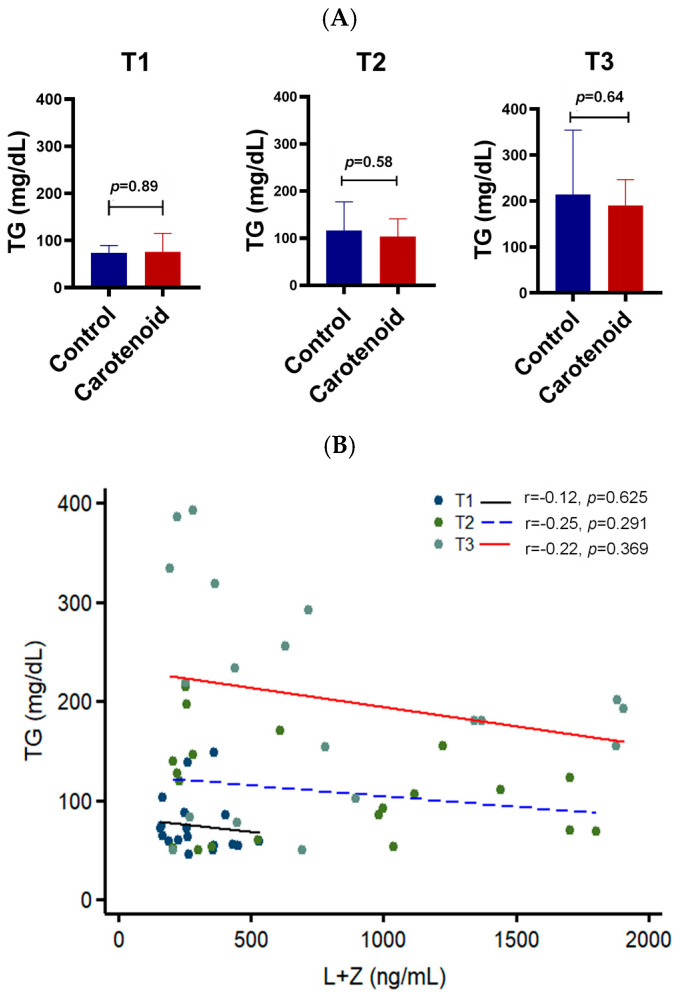
Effect of carotenoid intake on triglycerides in the human serum. Carotenoid intake reduces triglycerides. A reverse correlation is found between serum carotenoids and triglycerides. (**A**) Triglycerides (TG) in the serum of pregnant women. T1, first trimester; T2, second trimester; T3, third trimester. *n* = 10 subjects/group. (**B**) Correlation between serum carotenoids and triglycerides. T1, black solid line; T2, blue dashed line; T3, red solid line.

Triglycerides are the most common form of fat in the human body and are important indicators of cardiovascular and metabolic health. Triglyceride levels increased progressively throughout pregnancy, ranging from 15 to 200 mg/dL. Carotenoid supplementation was associated with lower triglyceride levels, although this reduction did not reach statistical significance (Figure 5). Correlation analysis showed inverse relationships between carotenoids and triglycerides, with *R*-values of −0.12, −0.25, and −0.22 across the three trimesters. Although these did not reach statistical significance, the consistent negative trend suggests carotenoid intake may reduce serum triglycerides.

Collectively, these findings indicate that carotenoid intake is positively associated with HDL cholesterol and inversely associated with triglycerides, although the correlations were not statistically significant in most pregnancy stages. Variability in serum carotenoid levels at enrollment likely contributed to this outcome. Another limitation of this study was the use of a mixed carotenoid supplement (10 mg lutein + 2 mg zeaxanthin), which was the only available formulation when the trial began.

To address these limitations and further assess the effects of individual carotenoids on HDL cholesterol elevation and triglyceride reduction, we measured serum lipid levels in macular carotenoid transgenic mice fed with zeaxanthin, lutein, β-carotene, or a carotenoid-free chow diet for one month. Zeaxanthin, lutein, and β-carotene were selected because they are the primary dietary carotenoids in human serum and retina.

**Figure 6:**
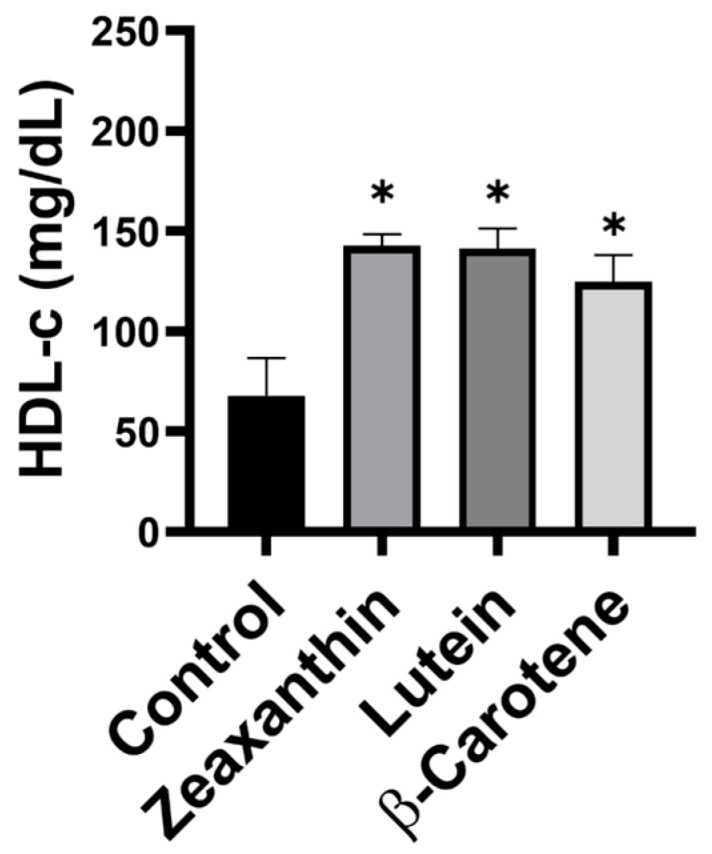
The level of HDL cholesterol in the serum of mice after carotenoid supplementation. The serum HDL cholesterol (HDL-c) is significantly increased in mice fed with retinal carotenoids lutein, zeaxanthin, and β-carotene for one month. *n* = 3/group; control, mice on placebo chow; * *p* < 0.05.

**Figure 7:**
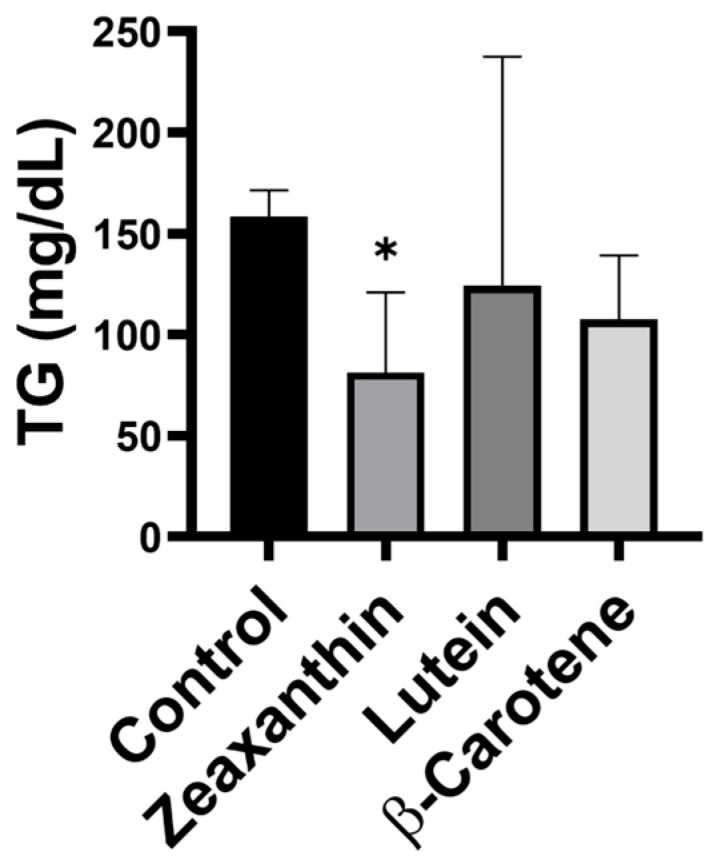
The level of triglycerides in the serum of mice after carotenoid supplementation. The serum triglycerides were reduced in mice fed with retinal carotenoids lutein, zeaxanthin, and β-carotene for one month. *n* = 3/group; control, mice on placebo chow; * *p* < 0.05.

Our carotenoid-feeding experiments showed that supplementation with zeaxanthin, lutein, or β-carotene significantly increased HDL cholesterol levels in mouse serum, nearly doubling them (Figure 6). Further analysis revealed that all three carotenoids were associated with reduced triglyceride levels; notably, only the zeaxanthin group exhibited a statistically significant 2.5-fold reduction (Figure 7). These animal data corroborate the clinical findings, demonstrating that carotenoid intake increases HDL cholesterol and reduces triglycerides, with zeaxanthin showing the strongest effects.

## 4. Discussion

To our knowledge, this is the first manuscript to integrate the interaction between carotenoids and the serum lipid profile. Data from our clinical trial and animal experiments demonstrate that carotenoid intake increases HDL cholesterol and reduces circulating triglycerides.

The mechanism underlying the elevation of serum HDL cholesterol induced by carotenoid supplementation is still not fully understood. However, this regulation appears to occur during cholesterol efflux from peripheral tissues to HDL particles, rather than through lipid exchange between HDL and LDL lipoproteins. HDL particles acquire cholesterol mainly through two pathways [37,38]. The first is reverse cholesterol transport, in which cholesterol is transferred onto HDL particles from peripheral tissues. The second is the CETP-mediated lipid exchange process, which transfers esterified cholesterol from HDL to LDL particles while returning triglycerides. In this study, we found that HDL cholesterol increased by approximately 1.2-fold after carotenoid supplementation in pregnant women for six months (Figure 3). However, no significant change was observed in LDL cholesterol (Figure 4), suggesting that carotenoids primarily influence cholesterol influx into HDL particles. Cholesterol is transported from peripheral cells to HDL particles through ABCA1 [37], an HDL receptor on the cell surface. Interestingly, a nonsense mutation in the *Abca1* gene of the Wisconsin chicken reduced not only serum HDL cholesterol but also retinal carotenoids [39]. This implies that ABCA1 may act as a scramblase, shuttling carotenoids into cells while exporting cholesterol out. This hypothesis warrants further investigation. Moreover, our animal experiments revealed that one month of supplementation with zeaxanthin, lutein, and β-carotene doubled HDL cholesterol levels in mouse serum—a stronger effect than six months of supplementation in human subjects. This may be attributed to the more controlled carotenoid content and higher relative doses in the mouse chow compared to the human diet.

The reduction in serum triglycerides induced by carotenoid intake appears to occur during the assembly of lipoproteins responsible for triglyceride transport. Chylomicrons and VLDL are the primary lipoproteins that carry triglycerides—chylomicrons transport dietary triglycerides from the intestine to circulation, while VLDL carries hepatic triglycerides to peripheral tissues [18]. Together, these two lipoproteins account for about 90% of serum triglycerides. Our data show that carotenoid supplementation led to an approximately 20% reduction in serum triglycerides (Figure 5), suggesting that carotenoids regulate triglyceride levels through effects on chylomicron or VLDL metabolism. Because human serum LDL cholesterol did not change significantly during carotenoid supplementation, the triglyceride reduction is unlikely to result from increased transport to peripheral tissues. LDL cholesterol and triglycerides are typically co-transported via the same cargo through endocytosis, suggesting instead that carotenoids regulate triglycerides in chylomicrons rather than VLDL. If so, this regulation likely occurs during the intestinal assembly of dietary triglycerides. In addition, our animal experiments demonstrated that zeaxanthin had a stronger triglyceride-lowering effect than lutein or β-carotene.

This study provides new insight into the selection of serum cholesterol fractions as biomarkers for human disease. Serum carotenoids are well-established biomarkers for several retinal conditions. We found that serum carotenoids were significantly associated with HDL cholesterol and triglycerides, but not with total cholesterol. This is expected, as total cholesterol reflects cholesterol across all serum lipoproteins, whereas HDL cholesterol and triglycerides show opposite correlations with carotenoids. These findings suggest that total cholesterol may not be an informative biomarker for human disease, particularly for retinal disease. Historically, total cholesterol was widely used because it was easy to measure; however, with modern analytical techniques, it is no longer the most accurate or meaningful indicator and should therefore be reconsidered.

Our findings demonstrate that carotenoid intake improves serum lipid profiles, suggesting a new protective role for retinal carotenoids. We and others have previously reported that lutein and zeaxanthin supplementation reduces light-induced retinal damage and lowers the risk of AMD, with zeaxanthin offering greater protection [40,41,42]. Here, we show that carotenoid supplementation increases HDL cholesterol—the “good” cholesterol known to protect against heart, liver, and brain diseases—suggesting that carotenoids may also have therapeutic potential beyond the retina.

Of course, this study has several limitations. The participants were pregnant women, whose lipid profiles may differ from those of men and non-pregnant women. In addition, it is important to note that the fasting conditions typically used to reduce variability in lipid measurements were not applied in this population, as fasting is generally not recommended during pregnancy. For consistency, fasting was also not implemented in our animal experiments. Lastly, we would like to point out that both the clinical trial and the animal experiments involved relatively small sample sizes. Studies with larger cohorts will be valuable for further strengthening our conclusions.

In conclusion, our data show that carotenoid intake increases HDL cholesterol and reduces triglycerides, thereby improving the serum lipid profile. These findings suggest that carotenoid supplementation not only benefits retinal diseases such as AMD but may also confer protection against other conditions, including cardiovascular disease, fatty liver disease, and Alzheimer’s disease.

## Data Availability

The original contributions presented in this study are included in the article. Further inquiries can be directed to the corresponding authors.
